# Characteristics of Posterior Root Tears of the Lateral Meniscus

**DOI:** 10.7759/cureus.115860

**Published:** 2026-09-06

**Authors:** Kamil Boguszewski, Lukasz Kopoczynski, Dorota Bartkowiak, Michal Wiesner, Lukasz Wodzinski, Bartlomiej Jakubczyk, Adam Lasota, Hubert Porazinski, Agnieszka Mlynarska, Oskar Ratajski

**Affiliations:** 1 Medicine, Nowy Szpital in Szubin, Bydgoszcz, POL; 2 Medicine, Szpital Morski im. PCK, Gdynia, POL; 3 Medicine, Dr Jan Biziel University Hospital No. 2 in Bydgoszcz, Bydgoszcz, POL; 4 Medicine, District Health Center Sp. z o.o. in Malbork, Malbork, POL; 5 Medicine, F. Ceynowa Specialist Hospital, Wejherowo, POL; 6 Medicine, Antoni Jurasz University Hospital No. 1 in Bydgoszcz, Bydgoszcz, POL; 7 Cardiology, Samodzielny Publiczny Szpital Wojewódzki im. Papieża Jana Pawła II w Zamościu, Zamość, POL; 8 Internal Medicine, Karol Marcinkowski University Hospital, Zielona Góra, POL

**Keywords:** knee injuries, lateral meniscus posterior root, meniscus tear, orthopedic arthroscopy, trauma and orthopedics

## Abstract

Lateral meniscus posterior root tears are increasingly recognized as clinically important injuries that can compromise meniscal function and alter knee joint biomechanics, particularly in the setting of anterior cruciate ligament (ACL) injury. This review synthesizes and critically evaluates the current literature on the anatomy, biomechanics, classification, epidemiology, diagnosis, and management of lateral meniscus posterior root tears, with consideration of similarities and differences compared with medial meniscus posterior root tears. Particular attention is given to the anatomical relationship of the lateral meniscus posterior root to the tibial plateau and adjacent structures, as well as to the biomechanical consequences of root disruption, including impaired load transmission, increased compartmental contact stresses, and potential effects on knee stability. Current classification approaches and diagnostic strategies are evaluated for their clinical applicability and limitations. The review further examines contemporary surgical management, focusing on arthroscopic anatomic root repair, repair techniques, patient- and injury-related factors influencing treatment selection, and the extent to which restoration of the native root attachment may improve meniscal function and knee mechanics. Available evidence regarding postoperative rehabilitation, complications, and clinical outcomes is also critically assessed. Particular consideration is given to the frequent coexistence of lateral meniscus posterior root tears with acute ACL injuries and the implications of an unrecognized or untreated root injury for joint stability and long-term meniscal preservation. By integrating anatomical, biomechanical, clinical, and surgical evidence, this review highlights current areas of consensus, limitations in the literature, and priorities for future research to improve diagnosis, treatment strategies, and patient outcomes.

## Introduction and background

Posterior meniscus root tears comprise a heterogeneous group of injuries whose clinical presentation and consequences depend strongly on the involved compartment and mechanism of injury. Posteromedial meniscus root tears are commonly associated with degenerative changes and are typically encountered in middle-aged or older patients, whereas lateral meniscus posterior root tears (LMPRTs) more often represent traumatic lesions associated with anterior cruciate ligament (ACL) injury [1,2].

This distinction has implications beyond patient demographics. Krych et al. demonstrated differences between medial and lateral root tears in injury presentation, associated pathology, treatment decisions, and surgical outcomes [3]. Accordingly, conclusions drawn from studies of degenerative medial root tears cannot be automatically extrapolated to traumatic lateral root injuries.

The meniscal roots represent the attachment sites of the meniscal horns to the tibial plateau, securing the menisci to bone and permitting axial loads to be transmitted into circumferential hoop stresses [1,2]. Loss of a functional root attachment may compromise this mechanism, but the magnitude of the resulting biomechanical disturbance is not identical for medial and lateral lesions. In the lateral compartment, tear completeness and preservation of the meniscofemoral ligaments (MFLs) influence the functional consequences of root disruption [4,5].

This review therefore focuses specifically on LMPRTs, with emphasis on features that distinguish them from medial root tears. Particular attention is given to diagnostic limitations, the interaction between LMPRT and ACL instability, criteria for repair versus observation, differences among repair strategies, and the available evidence regarding healing and clinical outcomes.

## Review

Anatomy

Knowledge of the lateral meniscus posterior root anatomy is particularly relevant when attempting anatomical repair. The posterior root of the lateral meniscus lies relatively close to its anterior root compared with the wider separation of the corresponding medial meniscal attachments. Anatomical studies describe the lateral meniscus posterior root as comprising an entheseal component at the tibial attachment, an obliquely oriented midsubstance component, and a junctional region continuous with the posterior horn [6,7].

Johannsen et al. provided quantitative landmarks for localization of the native attachment relative to the lateral tibial eminence, articular cartilage margin, and posterior cruciate ligament footprint [7]. Importantly, the attachment extends beyond the most readily visible central fibers and includes supplementary fibers that contribute to the overall footprint.

Rather than treating the root attachment as a single arthroscopic point, these observations support restoration of the broader native footprint during repair. This distinction is relevant because displacement of a tibial tunnel or anchor from the anatomical insertion changes the direction in which the repaired meniscus is tensioned and may limit restoration of its native mechanical function [1,7].

The lateral meniscus is further distinguished by its relationship with the MFLs. The ligaments of Humphrey and Wrisberg connect the posterior region of the lateral meniscus with the femur [4,5]. Consequently, two apparently similar LMPRTs may not produce equivalent functional impairment when MFL integrity differs.

Biomechanical consequences

The available biomechanical evidence indicates that the consequences of LMPR disruption depend on both the extent of meniscal injury and the integrity of secondary attachments.

Ode et al. demonstrated a progressive biomechanical effect as radial tear width increased. Partial tears produced relatively limited changes, whereas complete disruption substantially altered lateral compartment contact mechanics [8]. Schillhammer et al. subsequently showed that posterior horn detachment increased peak contact pressure and reduced contact area, with repair shifting these parameters toward the intact state [9]. Finite-element modeling by Bao et al. produced findings consistent with these experimental observations [10].

These studies should not, however, be interpreted as evidence that every LMPRT is functionally equivalent to complete meniscal loss. Forkel et al. demonstrated that preservation of the MFLs can mitigate changes in lateral compartment contact pressure following root disruption; additional loss of MFL function resulted in a considerably greater mechanical disturbance [11]. Preserved MFL integrity may partially maintain meniscal stability even in the presence of substantial root injury, whereas combined root and MFL disruption results in greater biomechanical impairment.

The relevance of the LMPR also extends beyond contact mechanics. Frank et al. identified the lateral meniscus posterior root and MFLs as secondary stabilizing structures in the ACL-deficient knee [12]. Subsequent cadaveric work similarly demonstrated changes in rotational and translational laxity after LMPR disruption and partial restoration following repair [13]. 

Taken together, these experiments support restoration when a lesion produces functional root incompetence, particularly in an ACL-deficient knee. They do not, however, establish that repair of every morphologically identified LMPRT improves long-term clinical outcomes or prevents osteoarthritis. Most mechanistic evidence derives from cadaveric, animal, or computational models, and this limitation should be kept in mind when translating biomechanical findings into treatment recommendations. Therefore, their direct clinical relevance and applicability in vivo remain to be established.

Classifications

Several classification approaches have been proposed for PLMRTs. West et al. described three principal tear patterns identified during ACL reconstruction [14]: Type I, root avulsion; Type II, radial split tear near the root insertion; and Type III, complex root tear with both radial and longitudinal components. Subsequently, Ahn et al. classified PLMRTs associated with ACL reconstruction into four patterns [15]: Type I, radial tear with an oblique flap; Type II, T-shaped tear consisting of radial and longitudinal components; Type III, longitudinal cleavage between the bony insertion and the MFL insertion; and Type IV, chronic inner-loss type characterized by substantial tissue loss between the meniscus and root.

Forkel et al. subsequently proposed a differentiated system incorporating MFL integrity [16]. Their classification distinguishes root avulsion, radial tearing close to the root with an intact MFL, and complete detachment of the posterior horn with loss of functional continuity. The inclusion of MFL status is relevant because these structures influence both contact mechanics and stability [4,5,11].

The existing classifications should therefore be regarded as complementary rather than interchangeable. The West and Ahn systems primarily characterize tear morphology, whereas the Forkel system incorporates an additional functional component. No current classification has been adequately validated as a predictor of healing, postoperative extrusion, reoperation, or patient-reported outcome. Consequently, classification alone should not determine treatment; morphology should be interpreted together with arthroscopic stability, tissue quality, reducibility, MFL integrity, and associated ACL injury.

Epidemiology

The association between LMPRT and ACL injury is one of the most consistent features distinguishing lateral from medial posterior root pathology. De Smet et al. identified a strong association between lateral meniscus posterior root tears and ACL disruption [17], while Brody et al. demonstrated an association between ACL injury, lateral root tearing, and lateral meniscal extrusion [18].

Clinical series have identified LMPRTs in a relevant minority of patients undergoing ACL reconstruction. However, prevalence estimates vary depending on the definition of root injury, study population, imaging protocol, and whether the diagnosis is based on MRI or arthroscopic inspection [15,19]. For this reason, individual prevalence estimates should not be treated as universally applicable.

More recent registry evidence provides additional information about the injury pattern. Lambrey et al. analyzed 5,359 patients undergoing ACL reconstruction and identified LMPRTs in 7.0% of cases. Male sex, revision surgery, low ACL-remnant volume, increased pivot-shift grade, and greater side-to-side laxity were associated with LMPRT [20]. Rather than demonstrating that these variables directly cause LMPRT, these findings suggest that lateral root injury may occur as part of a broader instability pattern associated with ACL disruption.

Diagnosis

Diagnosis of LMPRT requires a high index of suspicion because the clinical presentation is commonly dominated by the associated ACL injury. Joint-line pain, effusion, mechanical symptoms, and instability may occur, but none is sufficiently specific to establish the diagnosis.

MRI is the primary preoperative imaging modality, although its diagnostic limitations are clinically important. De Smet et al., using arthroscopy as the reference standard, demonstrated that LMPRTs can be identified on MRI but may be missed [17]. Relevant findings include direct root discontinuity or abnormal signal, a radial defect near the attachment, altered posterior horn morphology, sagittal loss of the normal meniscal appearance, and lateral meniscal extrusion [17,18].

Nguyen et al. further characterized MRI findings of lateral posterior root injury in children with ACL tears [21]. The importance of evaluating multiple findings rather than searching for a single pathognomonic sign is supported by Asai et al. In ACL-injured knees, individual MRI signs demonstrated limited sensitivity, whereas their combined assessment improved diagnostic performance [22].

This has a practical consequence: absence of a single characteristic MRI sign should not be used to exclude LMPRT. Likewise, absence of marked lateral meniscal extrusion does not establish root integrity. Preserved MFLs may restrict meniscal displacement even in the presence of substantial or complete root disruption; therefore, absence of extrusion does not exclude a clinically relevant LMPRT [11,18].

MRI findings should therefore be interpreted in the context of the injury mechanism and ACL status. During ACL reconstruction, careful arthroscopic inspection and probing of the posterolateral meniscal root may be particularly warranted when the injury mechanism, MRI findings, or intraoperative appearance raise suspicion of LMPRT. Arthroscopic assessment can establish whether the lesion is partial or complete, stable or unstable, reducible, and associated with loss of functional MFL support. These characteristics are directly relevant to treatment selection.

Treatment

Management of LMPRT should be based on the functional characteristics of the lesion rather than on the diagnosis of a root tear alone. Relevant factors include tear morphology and completeness, arthroscopic stability, tissue quality, reducibility, cartilage condition, MFL integrity, associated ACL instability, and patient activity level [1,19].

This approach helps distinguish two clinically different scenarios: a complete or unstable traumatic lesion that compromises root function and a stable lesion in which sufficient functional continuity remains. The former provides a stronger rationale for repair, whereas long-term observational data indicate that selected stable LMPRTs identified during ACL reconstruction can be managed without fixation [23,24].

Non-operative Treatment

Evidence supporting non-operative treatment of traumatic LMPRT is substantially less developed than the literature concerning degenerative medial root tears. Moreover, non-operative treatment should not be used interchangeably with leaving a stable lesion untreated during ACL reconstruction.

Shelbourne et al. reported long-term outcomes of selected lateral meniscus posterior root tears left in situ during ACL reconstruction [23]. Shumborski et al. subsequently evaluated stable LMPRTs left untreated at ACL reconstruction with a minimum 15-year follow-up. They found minimal long-term clinical detriment compared with patients with an intact lateral meniscus [24].

These findings do not justify the observation of a displaced or functionally incompetent root avulsion. Rather, they indicate that stability matters: a morphologically identifiable LMPRT with preserved functional continuity may behave differently from a complete unstable lesion.

Meniscectomy

Partial meniscectomy may remain necessary when tissue is irreparable or in selected knees with advanced degenerative changes, but it does not restore root function. Experimental studies demonstrate deterioration of lateral compartment mechanics when functional meniscal continuity is lost [8-10].

For traumatic LMPRT in otherwise repairable tissue, contemporary management therefore favors preservation rather than routine excision. This recommendation derives primarily from biomechanical principles and observational surgical outcomes rather than randomized comparisons between meniscectomy and repair.

Suture Anchor Repair

Suture-anchor fixation provides direct fixation of the meniscal tissue to the root footprint. Much of the historical literature describing this approach concerns medial rather than lateral posterior root tears [25-27], which limits direct extrapolation to LMPRT.

Familiari et al. described an all-suture anchor technique specifically for lateral posterior root repair [28]. Avoiding an additional transtibial tunnel may be advantageous when ACL reconstruction is performed simultaneously, although this remains primarily a technical rationale rather than evidence of superior clinical outcome.

Current comparative clinical evidence remains insufficient to conclude that anchor fixation provides superior healing or clinical outcomes compared with transtibial or side-to-side repair.

Transtibial Pull-Out Repair

Transtibial pull-out repair aims to restore the root to its tibial attachment by passing sutures through the meniscal tissue and securing them through a tibial tunnel [6,29]. Anatomical tunnel positioning is particularly important during combined ACL reconstruction because both procedures require tibial bone tunnels and poorly planned trajectories may interfere with one another.

Perry et al. systematically reviewed LMPR repair performed with ACL reconstruction. They found that repair improved joint contact mechanics in biomechanical studies, while most investigations assessing instability also reported improvement after repair. Clinical studies demonstrated postoperative functional improvement [30].

The evidence nevertheless does not establish the superiority of transtibial repair over other techniques. The clinical literature remains heterogeneous with respect to tear pattern, fixation method, outcome measures, and follow-up. Technique selection should therefore reflect lesion morphology and operative circumstances rather than assuming that a single fixation construct is universally superior.

All-Inside and Side-to-Side Repair

Not every LMPRT represents avulsion of the meniscus from its tibial footprint. Radial or longitudinal tears adjacent to an intact insertion may permit direct meniscus-to-meniscus fixation.

Ahn et al. reported favorable results following all-inside repair performed during ACL reconstruction [15]. Tear morphology should therefore influence repair selection. An avulsion-type lesion requires restoration of the meniscus to the tibial attachment. In contrast, a radial tear with an intact root insertion may be more appropriately treated with direct side-to-side fixation. In addition to tear morphology, repair selection should consider tear reducibility, tissue quality, MFL integrity, and the ability to achieve stable anatomical fixation.

Outcomes of treatment

Clinical studies generally report improvement following LMPR repair performed with ACL reconstruction, but the strength of this evidence should not be overstated.

Zheng et al. systematically reviewed nine studies involving 215 knees treated for radial or avulsed LMPR lesions during ACL reconstruction. Both side-to-side and pull-out repairs were associated with improved clinical outcomes, and second-look arthroscopy demonstrated a high rate of complete or partial healing after side-to-side repair. However, all included studies carried a high risk of bias [31].

Perry et al. reached broadly similar conclusions from combined biomechanical and clinical evidence [30]. Collectively, these studies support the repair of functionally important LMPRTs but do not demonstrate that repair is superior to observation across all tear patterns.

This distinction is reinforced by the long-term observations of Shelbourne et al. and Shumborski et al., in which selected stable tears left in situ did not result in substantial clinical deterioration [23,24]. The apparent discrepancy between these studies and the repair literature is therefore more likely to reflect differences in lesion stability and morphology than contradictory evidence.

Differences between lateral and medial root repair outcomes

Lateral and medial posterior root repair outcomes should not be compared without considering the substantially different populations in which these injuries occur.

Krych et al. demonstrated differences in presentation, treatment, and outcomes between medial and lateral root tears [3]. Khoo and Yau subsequently performed a systematic review and meta-analysis comparing medial and lateral root repair and identified more favorable structural and functional outcomes following lateral root repair [32].

More recent systematic evidence from Dzidzishvili et al. similarly reported higher healing rates, reduced postoperative extrusion, and more favorable clinical outcomes following lateral rather than medial posterior root repair [33].

These findings are clinically encouraging but do not establish that the lateral meniscus has an intrinsically greater capacity for healing. LMPRTs generally occur in younger patients after trauma, whereas medial root tears are more frequently associated with degeneration, cartilage damage, malalignment, and older age. Concomitant ACL reconstruction may also alter the biological and mechanical environment in which a lateral repair heals.

The available evidence therefore supports the more cautious conclusion that observed outcomes are generally more favorable after lateral root repair, rather than the stronger assertion that the lateral root necessarily has an intrinsically greater biological capacity for healing.

Recent comparative evidence is consistent with this interpretation. In a 2026 propensity-matched cohort analysis, Koukoulias et al. found that LMPR repair performed during ACL reconstruction resulted in postoperative stability and patient-reported outcomes comparable to those of matched patients undergoing isolated ACL reconstruction [34]. These results are encouraging, although further long-term studies, particularly with follow-up beyond 5-10 years, are needed to determine whether successful repair alters the risk of subsequent degenerative change.

Postoperative rehabilitation

Postoperative management following LMPR repair must balance protection of the healing meniscus against the risks associated with excessive immobilization, including stiffness and prolonged muscle inhibition.

Published rehabilitation strategies after combined LMPR repair and ACL reconstruction remain heterogeneous. In the systematic review by Benelli et al., 7 of 12 studies recommended non-weight-bearing status for at least four to six weeks, while 8 of 12 restricted knee range of motion to 0°-90° for four to six weeks. Brace use was recommended in 5 of 12 studies, further highlighting the substantial variability among published protocols [35].

The apparent discrepancy between these studies and the repair literature is therefore more likely to reflect differences in lesion stability and morphology than contradictory evidence. Current literature does not establish an optimal duration of restricted weight bearing or knee flexion following LMPR repair.

Postoperative progression should therefore account for the fixation construct, tear morphology, concomitant ACL reconstruction, additional cartilage or ligament procedures, and individual recovery. Earlier principles of meniscal repair rehabilitation, including gradual restoration of motion and progressive loading, remain relevant [36,37].

Complications

Complications of LMPR repair arise from both technical and biological factors. Technical problems include inaccurate tunnel or anchor placement, interference between the meniscal root and ACL tunnels, inadequate tissue capture, suture cut-through, and fixation failure. Postoperative problems include stiffness, persistent extrusion, incomplete healing, recurrent tearing, and the need for revision surgery.

Several traditional discussions of meniscal repair complications derive from studies of meniscal tears in general rather than LMPRT specifically [38-48]. Their applicability to contemporary root repair should therefore be interpreted cautiously. In particular, complications associated with older rigid meniscal implants have less relevance to current suture-based root fixation.

Age alone should not be treated as an absolute contraindication to repair. More clinically meaningful factors include cartilage degeneration, tissue quality, alignment, chronicity, activity level, and the ability to obtain stable anatomical fixation [38-40].

Interpretation of published failure rates is also complicated by inconsistent definitions. Some studies define failure according to symptoms or reoperation, whereas others use persistent extrusion, MRI evidence of non-healing, or second-look arthroscopy. These endpoints describe different aspects of treatment success and should not be combined without qualification.

For LMPRT specifically, persistent extrusion deserves particular attention. Structural healing and restoration of meniscal position should be considered distinct outcomes, as residual extrusion may persist despite evidence of root healing or clinical improvement. Consequently, healing status and postoperative meniscal extrusion should be evaluated separately when assessing treatment success.

Current treatment perspective

The available evidence supports a selective rather than uniform approach to LMPRT. Complete, displaced, or arthroscopically unstable traumatic lesions provide the strongest rationale for repair, particularly when encountered during ACL reconstruction [8-13,30,31]. Restoration of the root in this setting is supported by biomechanical evidence and clinical series demonstrating favorable healing and functional improvement.

Conversely, long-term observational evidence indicates that selected stable LMPRTs with preserved functional continuity may remain clinically well tolerated without fixation [23,24].

These findings are not necessarily contradictory. They suggest that the term LMPRT encompasses lesions with different biomechanical consequences. Treatment decisions should therefore incorporate morphology, completeness, arthroscopic stability, reducibility, MFL integrity, tissue quality, cartilage status, extrusion, and associated ACL instability rather than relying on the diagnostic label alone. In practical terms, complete or unstable lesions with loss of functional root continuity generally favor repair, particularly when anatomical reduction and stable fixation are achievable. In contrast, observation may be considered for selected stable lesions with preserved functional continuity.

## Conclusions

Lateral meniscus posterior root tears represent a distinct clinical entity that differs substantially from the more commonly degenerative medial meniscus posterior root tear. LMPRTs occur predominantly in the setting of traumatic ACL injury, and their functional consequences depend on tear morphology, completeness, stability, tissue quality, and preservation of the MFLs.

Diagnosis remains challenging because clinical findings are non-specific and individual MRI features may be subtle or absent. A combination of imaging findings, careful assessment of the injury mechanism, and systematic arthroscopic inspection provides the most reliable approach to identifying and characterizing these lesions.

Current evidence supports preservation of the lateral meniscus whenever feasible. However, the presence of an LMPRT alone should not be regarded as an absolute indication for surgical repair. Complete, displaced, or unstable lesions are more likely to benefit from anatomical restoration, whereas selected stable tears with preserved functional continuity may be managed without fixation. Treatment decisions should therefore be individualized according to tear morphology, arthroscopic stability, reducibility, MFL integrity, cartilage status, meniscal extrusion, associated ACL instability, and patient-related factors.

Several surgical techniques can be used depending on the lesion pattern, including transtibial pull-out repair, side-to-side or all-inside fixation, and suture-anchor repair. The available literature suggests generally favorable healing and functional outcomes after LMPRT repair, particularly when performed in conjunction with ACL reconstruction. Lateral root repairs also appear to demonstrate more favorable structural outcomes than medial root repairs. However, differences in patient characteristics, injury mechanism, cartilage status, and concomitant procedures must be considered when interpreting these comparisons.

Despite encouraging results, important uncertainties remain. Existing studies differ considerably in classification systems, indications for surgery, repair techniques, rehabilitation protocols, definitions of healing and failure, and follow-up duration. High-quality prospective studies with standardized diagnostic criteria, treatment algorithms, and outcome measures are therefore needed to clarify which lesions require repair, which surgical strategies provide the most durable restoration of function, and whether successful LMPRT treatment can reduce long-term cartilage degeneration and the development of osteoarthritis.

Overall, LMPRT should be regarded as a spectrum of injuries rather than a single, uniform condition, and management should be guided by the lesion's anatomical and functional characteristics rather than by the diagnosis of a posterior root tear alone.
